# Dietary Calcium Intake and Adherence to the Mediterranean Diet in Spanish Children: The ANIVA Study

**DOI:** 10.3390/ijerph14060637

**Published:** 2017-06-14

**Authors:** Nuria Rubio-López, Agustín Llopis-González, Yolanda Picó, María Morales-Suárez-Varela

**Affiliations:** 1 Unit of Public Health, Hygiene and Environmental Health, Department of Preventive Medicine and Public Health, Food Science, Toxicology and Legal Medicine, University of Valencia, 46100 Valencia, Spain; nuria.rubio@uv.es (N.R.-L.); agustin.llopis@uv.es (A.L.-G.); 2 Biomedical Research Center Network on Epidemiology and Public Health (CIBERESP), 28029 Madrid, Spain; yolanda.pico@uv.es; 3 Environmental and Food Safety Research Group (SAMA-UV), Faculty of Pharmacy, University of Valencia, 46100 Valencia, Spain; 4 Research Center on Desertification (CIDE, UV-CSIC-GV), Carretera Moncada-Náquera, 46113 Moncada, Spain

**Keywords:** calcium intake, nutrient intake, adherence, Mediterranean diet, children

## Abstract

The aim of this study was to evaluate the relationship of dietary calcium intake with anthropometric measures, physical activity and adherence to the Mediterranean diet (MedDiet) in 1176 Spanish children aged 6–9 years. Data were obtained from “Antropometría y Nutrición Infantil de Valencia” (ANIVA), a cross-sectional study of a representative sample. Dietary calcium intake assessed from three-day food records was compared to recommended daily intakes in Spain. Anthropometric measures (weight and height) were measured according to international standards and adherence to the MedDiet was evaluated using the Mediterranean Diet Quality Index (KIDMED) test. For the total sample of children, 25.8% had inadequate calcium intake, a significantly higher prevalence in girls (*p* = 0.006) and inadequate calcium intake was associated with lower height z-score (*p* = 0.001) for both sexes. In girls, there was an inverse relationship between calcium intake and body mass index (*p* = 0.001) and waist/hip ratio (*p* = 0.018). Boys presented a polarization in physical activity, reporting a greater level of both physical and sedentary activity in comparison with girls (*p* = 0.001). Children with poor adherence to MedDiet, even if they consume two yogurts or cheese (40 g) daily, adjusted by gender, age, total energy intake, physical activity and father’s level of education, are at risk of inadequate total calcium intake (odds ratio adjusted [ORa]: 3.36, 95% confidence interval [CI]: 1.13–9.94, *p* = 0.001). The intake of these dairy products was insufficient to cover calcium intake recommendations in this age group (6–9 years). It is important to prioritize health strategies that promote the MedDiet and to increase calcium intake in this age group.

## 1. Introduction

A healthy and balanced diet in children is essential to maintain and promote their health status. This is especially important for proper growth and development in children 6 to 9 years old [1,2]. Food consumption in this stage of life is affected by socio-cultural values, family’s financial situation and the media [3]. Recently, much concern has been voiced about whether Mediterranean countries are still adhering to the Mediterranean diet (MedDiet). Among various dietary patterns, the MedDiet has been accepted as one of the healthiest dietary models in the world [4,5]. Establishing healthy nutritional habits is important during childhood, since healthy nutrition practices established in this period often persist into adulthood [6]. The MedDiet pattern is represented by a diet pyramid characterized by common proportions of food consumption in the 16 countries around the Mediterranean Sea [7]. The typical MedDiet is characterised by high intakes of fruits, vegetables, pulses, whole grains (largely unrefined) cereals, tree nuts, as well as olive oil as the principal source of added fat, along with high to moderate intakes of fish and seafood, moderate consumption of eggs, poultry and dairy products (cheese and yoghurt), and low consumption of red meat [5,7,8,9]. This diet model, plays a preventive role in the development of cardiovascular and neurodegenerative diseases, certain cancers, diabetes and obesity [10,11]. Interestingly, good adherence to the MedDiet was positively associated with better academic performance in youths [8]. Despite the health benefits of the MedDiet, several studies have reported that dietary patterns in the Mediterranean region are changing to a more Westernised diet [11,12,13,14,15,16,17].

Nutrition is a well-known relevant factor for children’s growth and development [18,19,20]. Inadequate intake of energy or nutrients could have a detrimental effect on children’s health, especially in the formation of the bone structure [21]. Therefore, adequate calcium intake is important for growth [22,23,24,25,26] and optimum bone mass [27,28]. Long-term calcium dietary deficiency eventually depletes bone stores, which renders bones weak and prone to fracturing [29], and increases the risk of osteoporosis, which is one of the major public health problems in adulthood [24,30,31]. Bone mineral density is influenced by physical activity [32,33]. Rowlands et al. [34] found that bone mineral content was higher when both vigorous activity and calcium intake were greater, which led these authors to conclude that calcium intake and vigorous activity could have a synergistic effect on bone mass.

Unfortunately, a high proportion of children have inadequate calcium intakes, possibly due to an insufficient intake of dairy products [25,35] or other foods with high calcium content [36]. Food intake may vary among children due to social, cultural and economic factors, which affects dietary patterns [26,37,38]. Several studies have evaluated calcium intake in children [22,23,24,25,26,30,38] or adherence to the MedDiet in the Mediterranean population [12,13,14,15,16], but little is known about the relationship between calcium intake and adherence to the MedDiet. Therefore, the purpose of the study was to evaluate the relationship of dietary calcium intake with anthropometric measures, physical activity and adherence to the MedDiet in Spanish schoolchildren in Valencia.

## 2. Samples and Methods

### 2.1. Participants

“Antropometría y Nutrición Infantil de Valencia” (Valencian Anthropometry and Child Nutrition—ANIVA), a descriptive cross-sectional study, was conducted in 6–9-year-old schoolchildren who attended one of the 14 participating primary schools in the province of Valencia (Spain) [39]. Data collection took place during two academic years, 2013–2014 and 2014–2015. Children were selected by random cluster sampling in two stages: (1) schools were selected from lists provided by Regional Educational Authorities (i.e., public vs. private), this factor was used as an approximate indicator of socio-economic status; and (2) classrooms and pupils were selected.

Before the study was started, it was orally presented to the Board of Governors of each participating school. A letter was sent to the parents or legal guardians of all children invited to participate in this study. The letter explained the objectives and the tests that would be carried out and indicated that all the parents or guardians would need to give their written informed consent for their children to participate in the study. It was also indicated that collected data would be confidential according to Spanish personal data protection law. The study protocol complied with Declaration of Helsinki Guidelines and was approved by the Autonomic Secretary of Education, Generalitat Valenciana, Valencia, Spain (Ethics Committee 2014/29630).

For all children, the following inclusion criteria were applied: (a) between 6 and 9 years old; (b) enrolled at one of the 14 selected schools; (c) informed consent signed by the parent or guardian. The exclusion criteria were: (a) clinical diagnosis of chronic disease with dietary prescription; (b) absence from school on the days arranged to take body weight and height measures; (c) incomplete dietary records. The initial sample included 1432 children of both genders, of whom 11.38% declined to participate (*n* = 163). The subjects who provided incomplete dietary records (*n* = 61) or were absent on the day when anthropometric measurements were taken (*n* = 32) were removed and were not included in the study. The participation rate was 82.12% and the resulting final sample comprised 1176 children.

### 2.2. Examination Protocol and Measurements

#### 2.2.1. General Information

Parents or legal guardians were interviewed using a questionnaire to obtain information about child’s age, gender, medical history, medication, use of vitamin and mineral supplements, special dietary restrictions and food allergies. They were also asked about their level of education, and were classified as follows: no completed studies, primary school, secondary school, university studies or postgraduate studies. Level of education was classified into three levels: low, with no studies or with primary school; medium, with secondary school; or high, with university or postgraduate studies.

#### 2.2.2. Calcium Intake

To carry out the calcium intake evaluation, parents and legal guardians were asked to record all the foods and drinks consumed by their child over a 3-day period, including one non-school day (e.g., Sunday or Saturday) [40,41,42].

Parents/legal guardians were provided with written guidance on how to properly complete dietary records and estimate portion sizes using household measures (e.g., glass, cup, plates of various sizes, soup spoons, etc.). The same explanations were provided to the caregivers responsible for children in school dining halls. In addition, parents and caregivers were given an email and support telephone number, which they could use to help resolve any issues that arose while completing the diary. To assist in coding, parents were asked to provide food labels for foods eaten by the children, details about brands consumed, added ingredients and recipes for homemade dishes whenever possible.

The intake of calcium was calculated using the DIAL^®^ computer software, v 2.16 [43], which has been previously validated in Spain to assess diets and to manage nutritional data. The people responsible for the data input into the DIAL software from the food diaries are all Human Nutrition and Dietetics graduates. This open software includes a list of some of the enriched/fortified foods commonly available in Spain and other foods can be added to the database. With this feature, we were able to include the nutritional composition of packaged foods taken from food labels that parents and guardians submitted.

#### 2.2.3. Comparison with Dietary Reference Intakes

Calcium intake was assessed using the dietary reference intakes (DRIs) [44,45] proposed for the Spanish population. DRIs include estimated average requirement (EAR), the daily dietary intake level of nutrients considered sufficient by the Institute of Medicine Food and Nutrition Board [29] to meet the requirements of 50% of healthy individuals in each life stage and sex group. In this case, the EAR for calcium is 800 mg/day according to gender and age. Children were stratified according to calcium intake below the EAR or at or above the EAR.

#### 2.2.4. Assessment of Adherence to the Mediterranean Diet

Adherence to the MedDiet was assessed in school children using the Mediterranean Diet Quality Index (KIDMED) test completed by the children [46]. If any child did not understand the questions, they were helped by their teacher. KIDMED classifies individuals into three categories based on the KIDMED index calculated from participants’ answers. The index ranges from 0 to 12 and is based on a 16-question test (questions should be answered with “yes” or “no”). Questions inconsistent with the MedDiet were assigned a value of “−1”, and those consistent with the MedDiet were scored as “+1”. The sums of the values from the questionnaire were classified as follows (Table 1):

If the total score was ≥8, the child was considered to have good adherence to the MedDiet. If the score fell between 4 and 7, improvements had to be made to reach optimal MedDiet patterns (average adherence), and diet quality was very low (poor adherence) if their score was ≤3.

#### 2.2.5. Anthropometric Measurements

Children’s height and weight were measured during school hours by the same person following standard procedures described by the World Health Organization (WHO) [47], with children standing barefoot in light clothing. All the anthropometric measurements were taken twice and were averaged. Weight (in kg) was measured on a calibrated electronic load cell digital scale (OMRON BF511^®^, Tokyo, Japan) to the nearest 0.05 kg and height (in cm) was measured to the nearest 0.1 cm using a stadiometer (Seca 213^®^, Hamburg, Germany). With this data, body mass index (BMI) for age and gender (z-score), weight-for-age (z-score) and height-for-age (z-score), were calculated with the WHO Anthro software, v.3.2 (Geneva, Switzerland) [48]. Based on the obtained percentile ranking, BMI was used to classify children into one of the following four categories [49]: underweight (<5th percentile), normal weight (≥5th to <85th percentiles), overweight (≥85th to <95th percentiles) or obese (≥95th percentile).

Hip and waist circumferences were measured to the nearest 1 mm with a flexible inextensible tape by exerting slight pressure on the skin, but not compressing soft tissues [50]. The median of three measures of the same circumference was used for the analysis. The waist/hip ratio was then individually calculated in those children who were classified as obese, because this ratio is an indicator for cardiovascular and metabolic diseases only in obese children [51,52].

#### 2.2.6. Physical Activity and Sedentary Lifestyle

To assess children’s physical activity, parents were asked to report the number of days per week on which their children participated in physical activity and the duration in minutes of these [53]. Physical activity was classified into two levels [54]: adequate (in the case of at least 60 min of moderate to vigorous physical activity a day) and inadequate (in the case of less than 60 min of moderate to vigorous physical activity a day). In addition, parents were asked to report their children’s weekly frequency of sedentary activity, such as the hours spent watching TV, on the computer and playing video games. Sedentary activity was classified into three levels [53]: low (0 or <1 h/day), moderate (1–2 h/day) and high (≥2 h/day).

### 2.3. Statistical Analysis

Continuous variables were expressed as means (standard deviations, SD), whereas categorical variables were expressed as frequency (percentages, %). The Kolmogorov-Smirnov test was used to determine the normality of the distribution of the examined variables. Differences between means were established by a Student’s *t*-test (weight, height and BMI), and the non-parametric statistical test of the Mann-Whitney test was applied when the distribution of the results was not homogeneous (all the other continuous variables). The Chi-square test was used to compare differences between categorical variables. Multivariate logistic regression analyses were used to identify the possible risk factors of inadequate calcium intake with odds ratios (OR) and the corresponding 95% confidence intervals (CI) calculated. The OR of inadequate calcium intake based on the Spanish EAR was evaluated in relation to poor adherence to the MedDiet by taking good adherence to the MedDiet as a reference. The adjusted OR (ORa) for the main factors identified in the descriptive analysis was assessed and given for four models: (1) adjusted for gender; (2) adjusted for gender and age; (3) adjusted for gender, age, physical activity and total energy intake; (4) adjusted for gender, age, physical activity, total energy intake and father’s level of education.

All the *p*-values were two-tailed and statistical significance was set at the conventional cut-off of *p* < 0.05. Data was entered into a Microsoft Excel version 15.0 (Redmond, WA, USA), spreadsheet using a double-data entry to minimise the risk of errors. Data were then transferred to the IBM SPSS software, version 17.0 (SPSS Inc., Chicago, IL, USA).

## 3. Results

Basic characteristics stratified by gender are summarized in Table 2. A total of 25.8% of the sample presented inadequate calcium intake, with girls presenting a significantly higher percentage (29.1%) than boys (22.1%, *p* = 0.006). The height z-score showed that the schoolchildren with lower than recommended calcium intake were shorter in stature (*p* = 0.001), independently of gender. Statistically significant differences were found between obese children for the waist/hip ratio both in the whole sample (*p* = 0.001), and among boys and girls analysed separately (*p* = 0.001 and *p* = 0.018, respectively). When comparing within the same level of parental education (low, medium or high), a significant association with inadequate calcium intake was only found for boys with father’s with a low level of education (*p* = 0.035). Regarding physical activity, boys were more involved in physical activity than girls, independently of calcium intake (*p* = 0.001). However, when the comparison was made between children of the same gender but different classification of calcium intake, there was only significant difference in girls (*p* = 0.040). Concerning sedentary activities, time spent watching television, on the computer and playing video games were high for both genders, although higher for boys than girls. Statistically significant differences were found between girls and boys independently of calcium intake for the three levels of sedentary activity (low *p* = 0.001; moderate *p* = 0.039 and high *p* = 0.001). In general, children presented an average and good adherence to the MedDiet. Statistical differences were only found among girls for average adherence to the MedDiet (*p* = 0.043).

Consumption of foods with high calcium content is indicated in Table 3. As expected, consumption of dairy products for breakfast and 2 yoghurts/cheese (40 g) was high throughout the sample. Significant differences were observed in the consumption of dairy products for breakfast in girls (*p* = 0.007), therefore the consumption of dairy products for breakfast favors an adequate calcium intake.

Table 4 shows that poor MedDiet adherence does not significantly predict a deficiency in calcium intake: ORa: 1.65 (95%CI: 0.81–3.38). Children with poor adherence to the MedDiet were stratified according consumption of dairy for breakfast and two yogurts or cheese (40 g) during the day and various models were applied to adjust for demographic characteristics. It was observed that in children who consumed two yogurts or cheese (40 g) during the day, adjusted for gender, age, total energy intake, physical activity and father’s level of education, poor adherence to MedDiet is a risk factor for inadequate total calcium intake: ORa: 3.36 (95%CI: 1.13–9.94).

## 4. Discussion

The high prevalence of schoolchildren with low calcium intake was noteworthy and is of particular concern for public health because inadequate calcium intake in children may result in insufficient bone calcification, which could delay normal growth and lead to early onset of osteoporosis in later adulthood [54,55,56,57,58]. Several studies have investigated calcium intake in children in the Mediterranean region, Suarez-Cortina et al. [59] in a representative sample of 1176 Spanish children (5–12 years), found that calcium intake was lower than that recommended in 15.3% of the children. In contrast, Ortega et al. [24] indicated a higher prevalence of low calcium intake, 76.7% in Spanish children (7–11 years). In both studies, and in a paper by Serra-Majem et al. [60], prevalence of inadequate calcium intake was higher in girls than in boys. Only in Greek children [61] was calcium intake found to be higher in girls than in boys.

Concerning anthropometric measures, it was identified that children with inadequate calcium intake had a shorter stature, which is a similar finding to that reported in Bhargava [62] and Cao [63]. This supports the theory that calcium intake in this age group is important, especially for the formation of the bone structure. Similarly, to the results reported by Moreira et al. [64] and Castro-Burbajo et al. [65], the results showed an inverse relationship between calcium intake and the BMI z-score, but only in girls, probably because boys have more lean body mass and girls higher fat mass at this stage of life [66,67]. Some research [3,27,65] has indicated that calcium is involved in body weight regulation since its intake is associated with a drop in parathormone and 1,25-dihydroxy-cholecalciferol, which promote lower concentrations of intracellular calcium in adipose tissue and oxidation of fats rather than storage. Body weight is a highly multifactorial variable, so it is unlikely that a large fraction of its variability can be attributed to a single factor like calcium [64]. In addition to BMI, the waist/hip ratio is an accurate anthropometric indicator to be considered when estimating total body fat and intra-abdominal fat mass in obese subjects [51]. The results illustrate that there were significant differences in the waist/hip ratio when the comparison was made between children of the same gender and between girls versus boys when stratified by calcium intake. Obese girls with inadequate calcium intake have a higher risk of developing cardio metabolic overweight- and obesity-related diseases [51,52].

In the sample, the mother’s level of education did not present great differences; therefore, it seems it did not contribute significantly to adequate calcium intake. Nevertheless, a positive association between calcium intake and a low level of education in the fathers was identified in boys. This outcome contradicted the conclusions of Tornaritis et al. [68], who pointed out that the mother’s level of education had greater influence than that of the father’s on the child’s food consumption patterns. Along with the results of this study, Reicks et al. [69] indicated that parents play a key role in schoolchildren’s dietary patterns, as parental habits affect children’s eating behavior.

Physical activity is another important factor that may interact in the regulation of energy metabolism since it causes major perturbations in energy utilization [64], and together with sufficient calcium intake, plays an important role in proper bone development during growth, regardless of pubertal status [33,70]. Boys evaluated in this study participated in more organized physical activity than girls did, which is similar to that reported by Voltas et al. [5] and Wong et al. [55]. It was observed that boys also displayed more sedentary behaviour than girls did. An association was identified when all children were compared, for all three levels of sedentary activities.

The traditional MedDiet has been identified as a healthy diet and calcium intake is also related to a healthy diet [11]. Several authors [4,8] have used the KIDMED test to determine if subjects had good, average or poor adherence to the MedDiet. The results of this study agreed with those of other Spanish authors [5,15,71,72], who have demonstrated that about 50% of Spanish children and adolescents in other areas of Spain present good MedDiet adherence. The fact that girls have better adherence to the MedDiet than boys in this study is a reasonable finding because beginning in childhood, women are more preoccupied with body weight, which is more likely to affect their nutritional habits [73]. It was observed that when calcium intake was adequate, boys showed a slightly better adherence to the MedDiet than girls, similar to a Greek study [4]. However, of all the schoolchildren who reported inadequate calcium intake, girls showed better adherence to the MedDiet than boys did.

Consumption of dairy products is associated with a varied and balanced diet [74]. In contrast, the results of this study show a greater proportion of consumption of dairy products in groups where total calcium intake is lower than recommended. This can be interpreted as that children with poor MedDiet adherence seek to compensate their diet with dairy products, producing a relative but insufficient increase of total calcium intake. This is also possibly due to the low consumption of other foods with high calcium content, such as salmon, sardines, green leafy vegetables, nuts and fruit juices [36]. Although milk and dairy products are in terms of quantity and bioavailability, the best dietary source of calcium [24,26], an adequate calcium intake requires the consumption of various foods to cover intake recommendations. Therefore, in schoolchildren with poor adherence to MedDiet, and even if they consume two yogurts or cheese (40 g) daily, there is a risk of inadequate total calcium intake. Gender, physical activity and father’s level of education were some factors that affected the risk of inadequate calcium intake [15,75].

### Study Limitations and Strengths

This study is not without its limitations. Firstly, it is a cross-sectional study and, therefore, causality cannot be inferred. Secondly, this study covered a limited geographic area within Spain. Another limitation is the small sample sizes for the low adherence to the MedDiet.

A special strength of the present study is the fact that, to our knowledge, in the last decade no research has been carried out in Valencia concerning the relationship of calcium intake with anthropometric measures, physical activity and adherence to the MedDiet. This study offers strong internal validity given the low attrition rate obtained. 

The parentally reported information employed for the nutrition assessment in this age group is of good quality. Parents and schools were very interested in the study, and were extensively trained and supported to complete dietary records. Use of standardised procedures and validated instruments are also strengths of our study.

## 5. Conclusions

A high percentage of the children our sample, especially girls, did not meet the Spanish dietary recommendation for calcium intake. In general, low calcium intake was associated with a reduced height, low physical activity and poor adherence to the MedDiet when adjusting for confounding variables. Future nutritional interventions to promote the consumption of dairy products as well as other calcium-rich foods such as fish and green leafy vegetables, all within a MedDiet pattern, should be prioritized in order to favor the correct growth and development of children. A diet rich in milk and dairy products could be enough to cover recommendations, but at current consumption levels in our population, it was not enough to meet recommendations for many children.

## Figures and Tables

**Table 1:** KIDMED test to assess the Mediterranean Diet. (Modified from [46]).

KIDMED Test	Scoring
Takes a fruit or fruit juice every day	+1
Has a second fruit every day	+1
Has fresh or cooked vegetables regularly once a day	+1
Has fresh or cooked vegetables more than once a day	+1
Consumes fish regularly (at least 2–3 pieces/week)	+1
Goes more than once a week to a fast food restaurant (hamburger)	−1
Likes pulses and eats them more than once a week	+1
Consumes pasta or rice almost every day (5 times or more per week)	+1
Has cereals or grains (bread, etc.) for breakfast	+1
Consumes nuts regularly (at least 2–3 times/week)	+1
Uses olive oil at home	+1
Skips breakfast	−1
Has a dairy product for breakfast (yoghurt, milk, etc.)	+1
Has commercially baked goods or pastries for breakfast	−1
Takes two yoghurts or some cheese (40 g) daily	+1
Takes sweets and candy several times every day	−1

Notes: KIDMED: Mediterranean Diet Quality Index.

**Table 2:** Schoolchildren’s demographic characteristics according to gender and calcium intake.

Variable	Boys (*n* = 561, 47.7%)					Girls (*n* = 615, 52.3%)					*p*-Value Total ^*c*^
	Calcium Intake ≥ EAR (*n* = 437, 77.9%)		Calcium Intake < EAR (*n* = 124, 22.1%)		*p*-Value *^a^*	Calcium Intake ≥ EAR (*n* = 436, 70.9%)		Calcium Intake < EAR (*n* = 179, 29.1%)		*p*-Value *^b^*	
	M/*n*	SD/%	M/*n*	SD/%		M/*n*	SD/%	M/*n*	SD/%		
Age (years)	7.3	1.1	7.3	1.1	0.856	7.4	1.1	7.5	1.1	0.461	0.154
Weight z-score	1.2	1.3	1.1	1.5	0.384	1.0	1.2	1.1	1.1	0.698	0.343
Height z-score	0.9	1.4	0.7	1.3	0.118	0.9	1.3	0.7	1.3	0.157	0.001
BMI z-score	1.0	3.0	0.9	1.5	0.587	0.8	1.2	0.9	1.2	0.086	0.310
Waist (cm)	62.0	8.3	60.4	8.2	0.056	61.7	8.5	62.4	8.2	0.314	0.178
Hip (cm)	72.0	8.1	71.0	8.2	0.197	71.8	9.0	72.5	7.7	0.330	0.434
Waist/hip ratio *	0.89	0.05	0.86	0.05	0.001	0.88	0.05	0.89	0.04	0.018	0.001
Mother’s level of education (*n*, %)											
Low	132	30.2	43	34.7	0.343	131	30.0	65	36.3	0.129	0.353
Medium	148	33.9	41	33.1	0.867	136	31.2	54	30.2	0.802	0.768
High	157	35.9	40	32.3	0.450	169	38.8	60	33.5	0.222	0.450
Father’s level of education (*n*, %)											
Low	186	42.6	66	53.2	0.035	187	42.9	81	45.3	0.592	0.175
Medium	142	32.5	30	24.2	0.077	140	32.1	48	26.8	0.195	0.186
High	109	24.9	28	22.6	0.589	109	25.0	50	27.9	0.450	0.756
Level of physical activity (*n*, %)											
Inadequate	208	47.6	60	48.4	-	251	57.6	119	66.5	-	-
Adequate	229	52.4	64	51.6	0.876	185	42.4	60	33.5	0.040	0.001
Level of sedentary activity (*n*, %)											
Low	125	28.6	33	26.6	0.664	197	45.2	70	39.1	0.167	0.001
Moderate	208	47.6	61	49.2	0.754	171	39.2	85	47.5	0.059	0.039
High	104	23.8	30	24.2	0.927	68	15.6	24	13.4	0.489	0.001
Adherence to the MedDiet (*n*, %)											
Poor	23	5.2	11	8.9	0.137	17	3.9	12	6.7	0.136	0.139
Average	193	44.2	56	45.1	0.844	202	46.3	67	37.4	0.043	0.244
Good	221	50.6	57	46.0	0.365	217	49.8	100	55.9	0.169	0.366

Notes: BMI: body mass index; M: mean; *n*: number; SD: standard deviation; %: percentage; MedDiet: Mediterranean diet; EAR: estimated average requirement, *p* value < 0.05: considered statistically significant; *p*-value *^a^*: comparison between boys with calcium intake ≥ EAR vs. boys with calcium intake < EAR; *p* value *^b^*: comparison between girls with calcium intake ≥ EAR vs. girls with calcium intake < EAR; *p* value *^c^*: Comparison between all children; Calcium EAR: 800 mg/day; * Waist/hip ratio calculated only in obese children.

**Table 3:** Calcium-rich foods consumption.

Consumption	Boys (*n* = 561, 47.7%)					Girls (*n* = 615, 52.3%)					*p*-Value Total *^b^*
	Calcium Intake ≥ EAR (*n* = 437, 77.9%)		Calcium Intake < EAR (*n* = 124, 22.1%)		*p*-Value *^a^*	Calcium ≥ EAR (*n* = 436, 70.9%)		Calcium < EAR (*n* = 179, 29.1%)		*p*-Value *^a^*	
	*n*	%	*n*	%		*n*	%	*n*	%		
Dairy for breakfast/day	415	95.0	118	95.2	0.929	420	96.3	163	91.1	0.007	0.063
Two yogurts or cheese (40 g)/day	319	73.0	94	75.8	0.531	302	69.3	138	77.1	0.051	0.178

Notes: n: number; *p* value *^a^*: comparison between children of the same gender. *p* value *^b^*: comparison between all children independently of calcium intake. *p* value < 0.05 was considered statistically significant.

**Table 4:** Logistic regression model to predict the risk of inadequate calcium intake according to level of adherence to the MedDiet.

Adherence	Orc (95% CI) *p*-Value	ORa ^1^ (95% CI) *p*-Value	ORa ^2^ (95% CI) *p*-Value	ORa ^3^ (95% CI) *p*-Value	ORa ^4^ (95% CI) *p*-Value
Good adherence	1.00 (ref)	1.00 (ref)	1.00 (ref)	1.00 (ref)	1.00 (ref)
Poor adherence					
	1.51	1.56	1.66	1.61	1.65
	(0.80–2.92)	(0.80–3.02)	(0.85–3.25)	(0.80–3.26)	(0.81–3.38)
	0.229	0.027	0.025	0.001	0.001
Without two yogurts or cheese (40 g) consume	1.49	1.48	1.46	1.13	1.28
	(0.52–4.29)	(0.52–4.27)	(0.51–4.25)	(0.34–3.73)	(0.37–4.40)
	0.573	0.696	0.832	0.001	0.001
With two yogurts or cheese (40 g) consume	2.20	2.50	2.86	2.98	3.36
	(0.80–6.06)	(0.90–6.98)	(1.02–8.08)	(1.03–8.74)	(1.13–9.94)
	0.288	0.022	0.010	0.001	0.001
Without dairy for breakfast	2.55	2.39	2.84	1.64	18.76
	0.43–15.09	0.40–14.46	0.45–18.90	0.12–23.27	0.54–65.76
	0.304	0.529	0.314	0.041	0.012
With dairy for breakfast	0.92	0.78	1.15	0.960	4.14
	0.07–11.58	0.06–10.54	0.30–4.29	0.04–23.58	0.03–64.92
	0.946	0.632	0.811	0.957	0.431

Notes: ORc: crude odds ratio; ORa: adjusted odds ratio; CI: confidence intervals; ^1–4^: different adjustment models: ^1^ Adjusted for gender; ^2^ Adjusted for gender and age; ^3^ Adjusted for gender, age, physical activity and total energy intake; ^4^ Adjusted for gender, age, total energy intake, physical activity and father’s level of education; *p* value < 0.05: was considered statistically significant.

## Abbreviations

The following abbreviations are used in this manuscript:

ANIVA	Antropometría y Nutrición Infantil de Valencia
BMI	body mass index
CI	confidence interval
DRI	dietary reference intake
EAR	estimated average requirement
M	mean
MedDiet	Mediterranean diet
n	number
ORa	adjusted odds ratio
ORc	crude odds ratio
SD	standard deviation
WHO	World Health Organization
